# Clinician's Guide to Evaluating and Developing eHealth Interventions for Mental Health

**DOI:** 10.1176/appi.prcp.2020.20190036

**Published:** 2020-09-09

**Authors:** Steven J. Ondersma, Scott T. Walters

**Affiliations:** ^1^ Merrill Palmer Skillman Institute Department of Psychiatry and Behavioral Neurosciences Wayne State University Detroit; ^2^ Department of Health Behavior and Health Systems School of Public Health University of North Texas Health Science Center Fort Worth

**Keywords:** eHealth, mHealth, mental health, therapy, methods

## Abstract

**Objective:**

This review aimed to examine key information regarding technology‐delivered interventions for patients with mental health and/or substance use disorders and to provide support for efforts by psychiatrists and other mental health professionals in recommending applications or helping to develop new technology‐delivered interventions.

**Methods:**

The authors reviewed existing information about the appraisal, development, and evaluation of technology‐delivered interventions (eHealth interventions).

**Results:**

High‐level guidance is available for clinicians who want to evaluate eHealth applications for their patients. Clinicians should be familiar with existing models of eHealth intervention development and with traditional as well as unique elements in the evaluation of efficacy for these approaches. However, existing intervention development models have not been empirically validated, and only one includes empirical optimization as an inherent part of its process.

**Conclusions:**

Because of the proliferation of eHealth interventions, mental health professionals should bring to this area the same level of content knowledge, understanding of development and evaluation processes, and rigorous skepticism as they do for pharmacotherapy and therapist‐delivered behavioral interventions.

## HIGHLIGHTS

- eHealth interventions are rapidly transforming mental health treatment.
- Clinicians should apply the same rigor to evaluating eHealth interventions that they apply to pharmacological treatments or other behavioral therapies.
- High‐level guidance and recommendations regarding ideal eHealth intervention development are available to aid in such evaluation, but empirical evidence in support of these guidelines is lacking.

Software designed to promote mental health has many names. Common terms include eHealth (referring to any technology related to health), mHealth (the specific use of mobile devices for health promotion), behavioral intervention technologies, e‐interventions, e‐mental health, and computer‐delivered interventions. In the past decade, eHealth interventions for mental health and substance use have been a major focus of research and applied commercial efforts. Moreover, organizations such as the U.S. National Institute of Mental Health and the U.K. National Health Service recommend mental health apps to address the substantial gap between treatment need and treatment receipt. For instance, in 2017, 92.3% of people with a substance use disorder and 57.4% of those with any mental illness had not received any treatment during the past year (1). Moreover, eHealth interventions have some unique advantages because of their ease of use, low price, and high habit strength (2). They also have clear advantages in terms of remote delivery, a feature that has taken on added importance during the COVID‐19 pandemic.

These technologies have shown promising evidence, whether used as brief and highly focused interventions for substance use (3, 4) or as extended interventions for a range of conditions, including depression (5, 6, 7, 8), suicidality (9), anxiety (7, 10, 11, 12, 13), sleep and eating disorders (14), and substance use disorders (15, 16). The National Institute of Mental Health (17) classifies mental health apps in terms of their basic approach to addressing mental health: self‐management, improvement of cognition, skills training, supportive care, symptom tracking, and passive data collection. However, these findings do not suggest that all technology‐delivered interventions are equal. Psychiatrists and other mental health professionals have a critical role as gatekeepers of the more than 1,400 mobile apps designed to promote mental health (18). Although the U.S. Food and Drug Administration (19) is working toward a regulatory model for digital health applications (called the Digital Health Software Precertification Program), this effort is in its early stages and is initially focusing only on software (20) that is used as a medical device (i.e., designed for medical purposes, such as viewing images from magnetic resonance imaging or for diagnosis of a condition). Finally, psychiatrists and other mental health professionals may also be included as key members of teams seeking to develop new technology‐based interventions for mental health.

To perform this function, mental health professionals sometimes rely on professional conferences to gain knowledge about current trends in their field. In a review of technology‐related offerings at mental health conferences from 2013 to 2015, East and Havard (2) estimated that only 4.1% (N=179 of 4,404) of sessions were technology‐related, and only 0.3% addressed mental health apps. The authors concluded that professional conference training lags significantly behind the available offerings, and that professional associations should increase their opportunities for clinicians to learn more about the ways that technology can be used to improve mental health treatment. This call is even more urgent because apps often make claims of effectiveness that are not supported by scientific literature (18).

In this review, we aim to provide an overview of what is known about the evaluation of eHealth interventions, which encompass computer‐delivered as well as mobile interventions. Although the term “eHealth intervention” can refer to interventions addressing any health‐related behavior, we focus on those addressing mental health and substance use. We first address the initial factors to consider in evaluating an eHealth application, much of which will mirror information already widely available, including in an online guide from the American Psychiatric Association (APA) (21). We then focus on the current state of the art in the development and testing of eHealth technologies, from early stages through efficacy and effectiveness testing. As a high‐level overview, this review is intended to help orient mental health professionals to key considerations in the evaluation of eHealth interventions for clinical use or when serving as an informed member of an intervention development team.

We focus on three broad categories that encompass most of the available eHealth interventions for mental health. The largest category consists of mHealth applications, most of which are available through the Apple App Store (for iPhones, iPads, and the Apple Watch) or the Google Play App Store (for devices running the Android operating system). We also include text messaging applications, some of which are stand‐alone and others of which are incorporated into mHealth apps. Finally, we include interventions that are described as Web‐based (or Web‐delivered) or Internet‐based (or Internet‐delivered), some of which are also available as a mobile app. This last category includes many of the available multisession interventions for depression, anxiety, substance use, or sleep problems that are accessed via a Web site.

## OVERVIEW OF EHEALTH INTERVENTION CURATION

As the number of eHealth interventions has risen, so have resources to help physicians use these tools (22, 23, 24, 25). One example is the APA's app evaluation model (21), shown in Box 1. According to the APA model, the decision to recommend an app should be based on several factors. Not all clients benefit from the same approach; some patients may benefit from a program that draws from cognitive behavioral therapy, and other patients may benefit from a program that uses motivational feedback or monitoring. Because of these differing needs, clinicians should consider a range of information in helping patients select an app as part of their treatment plan. At each step, the clinician gathers information to decide whether that criterion is met. If an app fails one of the steps, the clinician would not proceed to the next step (and would probably not recommend the app to the patient). Although there is no minimum number of items an app must meet to be considered good, one would expect that better apps would meet more criteria. It is certainly possible that a client may decide to use an app that meets fewer criteria. In that case, the missing items may present opportunities for discussion, or at least may be shortcomings that the client should be aware of when using the app. Alternately, Boudreaux and colleagues (24) suggest an approach to overall mHealth application evaluation that involves first checking the scientific literature, followed by searching other sources, such as app stores and reviews, and subsequent pilot testing and elicitation of feedback from the patients participating in the tests. Other guidance is available for general integration of behavioral health apps into practice (13) as well as in the use of mobile apps for suicide prevention (26).

**BOX 1. American Psychiatric Association App Evaluation Model**

Step 1: Gather background information before evaluating the app

- Who is the developer?
- What is the cost?
- On what platforms does it operate?

Step 2: Understand app security and privacy

- What data are collected and shared?
- Can you delete or opt out of data collection?
- Are data encrypted? HIPAA compliant?

Step 3: Determine whether the app is likely to be effective

- After trying out the app, does it actually do what it says it does?
- Is there peer‐reviewed evidence to support the approach?
- Do the user ratings suggest that people find the app helpful?

Step 4: Consider ease of use

- Will the client be able to use the app with minimal assistance?
- Does it need an active connection to the Internet?
- Is it customizable? Accessible? Culturally relevant?

Step 5: Understand the extent to which the app is interoperable

- Can the app share information with other electronic health record systems or data tools?
- Can the data be exported? Printed out?

Adapted from the American Psychiatric Association's App Evaluation Model.

The literature on eHealth interventions suggests that steps 3 and 4 of the APA's app evaluation model (Box 1) deserve special attention. Despite the encouraging evidence regarding efficacy cited above, technology‐delivered interventions consistently show low rates of engagement and retention. For example, when two large health care systems sent letters to patients who smoke to inform them of the availability of an Internet‐based eHealth intervention for smoking and also engaged in widespread advertising, only 7% of the patients visited the study Web site, and less than 3% enrolled in the program (27). Among those who begin eHealth interventions, many fail to engage in even minimal use and even more fail to complete the intervention (28); one review (29) reported completion or sustained use rates of 0.5% to 28.6%. In response, experts have suggested that apps be developed with much greater attention to the needs and preferences of the end users (patients) rather than basing content only on what experts believe to be best (30, 31).

Other more direct guidance is also available. For example, the United Kingdom's National Health Service (32) provides a curated library of apps that have met a range of standards, including outcome evidence, usability, safety, and data security. The products included must be easily available and demonstrate security, privacy, and clinical efficacy. A commercial Web site (www.ourmobilehealth.com) offers a similar service for providers willing to pay for apps specifically designed to fit their practice. The site describes a review process to identify apps that are “relevant, trustworthy, and engaging.” The site also provides help to developers. For instance, parties who submit apps for consideration can receive feedback on changes that might improve usability, interoperability, privacy, and safety. When the app has met the site's requirements, it is added to the curated library. Similarly, a nonprofit association (www.psyberguide.org) (33) also provides ratings of eHealth interventions on a range of factors; many apps available on this site also include a formal expert review in addition to the ratings.

In summary, an initial app evaluation entails gathering information about accessibility, focus, security, user ratings, and whether the app has been endorsed by any credible organizations. However, although these first‐pass factors provide useful guidance, they are inevitably a superficial approach to a deeply complex process. Evidence of state‐of‐the‐art development and efficacy in affecting one or more well‐defined health outcomes are critical to an in‐depth evaluation of any mental health app.

## BEST PRACTICES IN APP DEVELOPMENT AND EVALUATION

To merit regular promotion in health care settings, eHealth interventions should be subject to the same rigorous safety and efficacy testing as therapist‐delivered interventions. This section provides an overview of best practices in the development and evaluation of eHealth interventions for mental health as well as an evaluation of the overall state of the science and recommendations for future progress.

### eHealth Intervention Development Models

A number of overlapping theoretical and procedural models are available to guide the development of eHealth interventions. Developers may borrow from several of these at once or may develop their applications without any systematic guiding theory or model. Interventions that are informed by one or more theories, however, tend to be more effective than those that are not (34). Although this review will not touch on specific theories of behavior change, such as the theory of planned behavior (35) or self‐determination theory (36), we review several of the most influential intervention development models.

#### Intervention mapping

Intervention mapping (37) is a framework designed to guide development of health promotion interventions. It was developed as a response to the lack of structure in health promotion program development. It seeks to explicitly connect theory and prior findings to the intervention development process. It does so through development of an initial model of the problem, followed by a detailed logic model of how change will be effected, using theory‐based intervention approaches that are organized into a coherent whole that will maximize implementation and scalability. Intervention mapping is an influential and commonly used approach that promotes rigorous connections between the identified problem and intervention design, with thorough integration of theory and the available literature. For example, Brendryen and colleagues (38) used intervention mapping to develop an online alcohol intervention. The goal of the intervention was to help clients drink less by developing self‐regulation skills to maintain change over time and adjust their coping during moments of risk. The authors used strategies such as screening with brief personalized feedback, content “tunneling” (i.e., delivering content in a fixed sequence, thereby reducing user burden), goal setting (i.e., having clients determine drinking goals ahead of time and report on those goals), and just‐in‐time therapy via prerecorded audio dialogue. The authors suggested that the clear program blueprint makes the intervention more coherent and will help clarify results from future program evaluations.

#### Behavioral intervention technology (BIT)

BIT is a relatively recent model that, unlike intervention mapping, was designed specifically to inform development of eHealth interventions (39). The BIT model combines technological elements and principles of behavior change and integrates the evaluation approaches typically taken by software developers with those of behavioral science. Broadly, the BIT model asks developers to specify why (specific aims such as reducing depression), conceptually how (specific behavior change strategies, such as motivation enhancement), what (specific technical elements, such as tailored messaging), technically how (e.g., technical platform, design), and when (frequency and timing of the intervention). This framework is then integrated with a technological framework, referred to as BIT‐tech, designed to guide software developers. BIT‐tech is composed of four components: the profiler (who collects data to define the user and the environment), the intervention planner (who specifies the exact intervention element for each timepoint), the intervention repository (which stores all available intervention components), and the user interface (the front end that presents a specific intervention element to the user at a particular time).

Ranney and colleagues (40, 41) used the BIT model to develop an 8‐week texting intervention to reduce violence and depression among adolescents being discharged from the emergency department. The tailored text messages reinforced cognitive reappraisal, emotion self‐regulation, and the use of self‐efficacy skills. On the basis of prior work, the developers created three message groups: one for girls with low‐violence behavior, one for boys with low‐violence behavior, and one for adolescents of both genders with high violence behavior. Participants received daily messages that were based on their group and their current mood rating. They could request additional messages on demand. The three groups received similar message content that differed in terms of behavioral activation activities, types of stressors, and language used in the messages.

#### Person‐based approach (PBA)

An alternative approach to eHealth intervention development was offered by Yardley and colleagues (42). They called this approach “person centered” to emphasize the perspectives of the end users in the development of the intervention. In the PBA, a wide range of consumers provide perspectives on what parts of the program will be used, by whom, and in what context. The PBA focuses on two key steps: in‐depth qualitative research with representative end users that begins early in the development and continues throughout all stages, including implementation of the intervention; and identification of guiding principles that inform the selection of behavior change techniques as well as choices about how the technology will embody those techniques. The PBA is a sequential and practical model that emphasizes continuing input from end users to inform development and implementation. For instance, Yardley and colleagues (42) provide an example of a common intervention feature that was rated poorly by a group of end users. Mobile health interventions sometimes use a person's location to send tailored messages. However, end user focus groups tended to be skeptical about this strategy, believing that the location‐sensing was sometimes inaccurate or misconstrued and that location‐triggered messages might sometimes have an iatrogenic affect (e.g., by reminding the person that he or she is at risk).

#### Multiphase optimization strategy (MOST)

The MOST approach defines three key phases of intervention development: preparation, optimization, and evaluation (43, 44). In the preparation phase, the MOST approach uses activities that are similar to those in intervention mapping and BIT, for instance clarifying the aims of the intervention, gathering relevant theoretical and empirical information from the literature, and developing a theoretical model. However, the MOST approach goes beyond other models, applying engineering approaches to evaluate components of the intervention that were identified during the preparation stage, typically by using one or more fractional factorial trials. The logic for these factorial trials is that engineering and design comprise too many options to be able to test out every possible combination. Thus, the developer identifies key components and tests the different options, often by using lower scientific standards for group assignment (e.g., convenience assignment), size (e.g., fewer subjects), and statistical analysis (e.g., effect sizes rather than statistical significance). The goal is to generate the most effective prototype without testing every possible combination. Collins and colleagues (43) give an example of using the MOST approach to develop a smoking cessation program that contained theoretically derived units, such as outcome expectation messages, efficacy expectation messages, message framing, testimonials, exposure schedule, and message sourcing. The design process would help to decide which components to focus on and the amount of content in each unit. This optimization stage is followed by an evaluation—in a traditional randomized trial—of an intervention consisting of the strongest subset of components. The MOST approach is unique from the models described thus far, in that it incorporates empirical evidence (again, largely gathered through factorial trials) as a key step between intervention design and testing in a randomized trial.

#### Additional early design methods

Although not specific to the design of eHealth interventions, the user‐centered design (UCD) approach (45) is a frequent part of the broader intervention design models reviewed above. UCD puts user needs at the center of the software design process, from its earliest stages and throughout development. UCD maintains a consistent focus on who will be using the intended software, for what reasons, and in what context to ensure that the final product fits the context and meets the needs of its target audience. eHealth intervention apps that have incorporated UCD from the beginning may be more likely to be seen by patients as relevant, easy to use, and intuitive, in part because of careful analysis of the users' needs and the contexts in which they might use the technology.

Similarly, although not a design approach in itself, the behavior change technique taxonomy (46) has become an important resource for many eHealth intervention designers. This taxonomy was designed to create a common language for describing behavioral intervention techniques, in part through input from a broad range of experts who sought to define and cluster all known approaches. The current taxonomy consists of 93 discrete behavior change techniques arranged under 16 clusters. For example, cluster 1, scheduled consequences, includes techniques such as differential reinforcement and shaping, and cluster 8, feedback and monitoring, includes techniques such as biofeedback and self‐monitoring of behavior.

#### Summary of eHealth intervention development models

The models discussed above are well known in the field, and thus it is important to be aware of them when evaluating or developing eHealth interventions. However, four points should be highlighted with respect to these models. First, although all of them can provide important guidance in intervention development, they vary widely in terms of level of guidance and detail. Second, these approaches offer only a framework; the difficult work of designing the best possible intervention, with incomplete guidance from an imperfect literature, remains. Third, none of these models has been tested with the level of rigor needed to conclude that their use will lead to a more effective intervention. In fact, only the MOST approach includes empirical evaluation as part of the development process. Fourth, although these approaches include a great deal of expert advice regarding intervention development, there is certainly additional general advice to be found. For example, Michie and colleagues (47) synthesized the consensus of eHealth intervention experts regarding the development and evaluation of eHealth interventions. Many of their recommendations (e.g., adopt engineering methodologies, such as factorial trials; use person‐centered and qualitative approaches throughout the development process; and link theory to behavior change techniques) are reflected in the above models. However, many other of Michie et al.'s recommended best practices (e.g., use Bayesian approaches to improve predictive modeling capabilities, evaluate cost‐effectiveness, ensure compliance with national standards) can be characterized as general advice that should be considered regardless of the development framework being used.

### eHealth App Evaluation

Software development evaluation relies on metrics such as usability, growth and patterns in use, and sustainability of use. These metrics also apply to eHealth interventions. However, eHealth interventions, like other behavioral interventions, must also demonstrate efficacy. Some makers of eHealth interventions highlight superficially impressive statistics regarding efficacy, which may be accompanied by references to studies in peer‐reviewed journals. However, as with any claim, significant skepticism is warranted. Below we turn to methods for evaluating the efficacy and/or effectiveness of eHealth interventions.

#### Single‐case designs

Single‐case research designs (also called single‐subject, small‐N, or N‐of‐one designs) are dramatically underused (48), despite having multiple advantages—particularly during the early stages of eHealth intervention design (49). Although they do not allow the causal inference afforded by randomized trials, these approaches can strongly suggest causality and as such are included as evidence in some evaluations of whether an intervention meets criteria for being evidence‐based (e.g., the Federal What Works Clearinghouse) (50). In their simplest form, single‐case designs involve systematic provision and removal of an intervention along with continuous assessment in an attempt to identify associated fluctuations in an outcome. For example, if a study participant reliably shows higher fruit and vegetable intake on days when she receives multiple text‐message reminders compared with days on which those reminders are not provided, the efficacy of those reminders is supported. This evidence is stronger if it remains true across multiple on‐off trials with irregular schedules (e.g., 4 days on, 2 days off; 2 days on, 1 day off). Similarly, if the pattern holds true with four additional participants, the efficacy of the reminders would be further supported.

Multiple baseline designs are an alternate single‐case approach. In this approach, two or more behaviors are targeted (e.g., depression, smoking cessation), but an intervention is introduced for one behavior at a time to see if the change in each outcome will coincide with introduction of the intervention targeting that behavior. Multiple baseline designs are powerful ways to provide evidence of causality but are most appropriate when multiple discrete behavioral outcomes can be addressed sequentially and when a stable baseline can be demonstrated prior to the introduction of the eHealth intervention. Regardless, this and other single‐case or small‐N designs are valid approaches that should not be overlooked. See Dallery et al. (49, 51) for a full discussion of how single‐case designs can be used to evaluate eHealth interventions.

#### Interrupted‐time‐series designs

Extending the single‐case or small‐N approach to larger samples can also yield powerful evidence in support of eHealth interventions, even without the use of a control condition. Interrupted‐time‐series approaches seek to imply causality through showing a disruption in a stable baseline that coincides with introduction of an intervention (52). Thus, a simple single‐group pre‐post design (AB, in which A is the pretest measure and B is the posttest measure) is extended to include multiple pre‐ and postintervention observations (e.g., AAAAABBBBB). If an otherwise stable baseline value suddenly changes after introduction of the intervention, and if that change is sustained throughout multiple postintervention observations, then the results strongly suggest that changes in the outcome measure can be attributed to the intervention. The stepped‐wedge design (53, 54) is an extension of this approach in which individual interrupted‐time‐series studies are conducted separately within multiple discrete sites (such as outpatient clinics or schools). If an eHealth intervention is introduced at those sites in a staggered way, with order being randomly selected, and if a change in the outcome of interest is consistently demonstrated following introduction of the intervention (but not before), then there is strong evidence for causality.

#### Randomized trials

As with other interventions, randomized trials remain the gold standard for evaluation of the efficacy and effectiveness of eHealth interventions. However, as is increasingly being highlighted (55, 56), the rigor of randomized trials is highly variable. Important factors include preregistration of trials, consistency of primary outcomes and analytic methods with those outlined during preregistration, use of intent‐to‐treat analyses, adequate sample size, control for multiple comparisons, use of reliable and valid measures, blinding of evaluators, and evaluation of potential bias. A rigorous trial should also use appropriate methods to address attrition, such as multiple imputation or full information maximum likelihood techniques as opposed to listwise deletion (simply dropping cases with missing outcome data), last observation carried forward (using data from the last available observation), or presuming failure among those lost to attrition (57, 58).

Choice of control and/or comparison group is also a key design decision, with each option carrying a unique set of pros and cons. Disagreement regarding this choice, in particularly as applied to eHealth interventions, has been significant enough that the National Institutes of Health (59) formed an expert panel to develop a framework for selection of comparators in trials of health‐related behavioral interventions. That panel developed the pragmatic model for comparator selection in health‐related behavioral trials, which outlines that selection of comparators should take into account the purpose of the trial (i.e., whether the goal is to identify whether the intervention has an effect of any kind, how that effect compares with the current standard of care, or how and/or why the intervention works); the developmental stage of the intervention (e.g., early or late); and the research context (e.g., the availability of alternative services in a given area) (59). For example, although an initial study of an eHealth intervention may use a very minimal comparator, such as a waitlist control or other inactive control condition, later studies may require a more formidable comparator, such as an intervention delivered by a therapist.

Randomized trials of eHealth interventions carry other unique considerations as well. For example, Mohr and colleagues (60, 61) have highlighted some of the reasons the rapidly changing landscape of computer‐based intervention technologies is not suited to the typical model of conducting multiple years‐long trials of a particular intervention. In a traditional model, the intervention being tested is a medication or a therapeutic approach that changes little or not at all, either in its own makeup or in how it is altered to fit a particular setting or sample. In contrast, part of the advantage of technology is that it evolves rapidly as new information or needs are identified. Mohr and colleagues (62) suggested a focus on hybrid implementation‐efficacy trials that are designed specifically to address the needs of a particular setting (30) and testing of intervention principles rather than a specific, locked‐down intervention.

## Discussion and Conclusions

Technology is rapidly transforming health care, including mental health treatment. Future technology could reshape mental health care in critical ways that bring great promise as well as new challenges (63). Mental health professionals must become knowledgeable about the evaluation of these tools and about their integration into ongoing care (24). As discussed above, guidelines for evaluating eHealth interventions, curated and/or recommended app lists, growing involvement by the Food and Drug Administration, and an increasing evidence base are available to support providers and health care systems in this effort. Furthermore, the skills needed to critically evaluate the evidence base in this area are similar to those needed to evaluate medications or traditional behavioral interventions (although providers should be aware of important differences as well, including the potential importance of designing software with input from patients and the greater fluidity of technology‐based approaches). Experts in this area have developed guidelines to inform the entire process and have suggested that use of these guidelines can result in a higher‐quality product. Empirical evidence in support of these development models is lacking, however; no clear data suggest that app development following these recommended guidelines results in more efficacious or scalable interventions.

The overall landscape of technology‐based tools is such that some (63, 64) have suggested the development of a new type of health care professional called a “technology specialist,” who could serve as a resource for patients and providers in recommending applications and promoting their sustained use. Even if such a role should become commonplace, it is ultimately the clinicians who must direct care for their clients. Mental health professionals who are knowledgeable and skilled regarding technology will be prepared to take on this important role.
